# AmpC hyperproduction in a *Cedecea davisae* implant-associated bone infection during treatment: a case report and therapeutic implications

**DOI:** 10.1186/s12879-021-07000-y

**Published:** 2022-01-06

**Authors:** Julia Notter, Salome N. Seiffert, Maria Zimmermann-Kogadeeva, Anja Bösch, Robert Wenger, Carol Strahm, Manuel Frischknecht, David M. Livermore, Baharak Babouee Flury

**Affiliations:** 1 Division of Infectious Diseases and Hospital Epidemiology, Kantonsspital St. Gallen, St. Gallen, Switzerland; 2 Division of Human Microbiology, Centre for Laboratory Medicine, St. Gallen, Switzerland; 3 Genome Biology Unit, European Molecular Biology Laboratory (EMBL), Heidelberg, Germany; 4 Medical Research Centre, Kantonsspital St. Gallen, St. Gallen, Switzerland; 5 Division of Hand, Plastic and Reconstructive Surgery, Kantonsspital St. Gallen, St. Gallen, Switzerland; 6 Norwich Medical School, University of East Anglia, Norwich, UK

**Keywords:** *Cedecea davisae*, AmpC, Hyperproducing, Cefepime, Resistance evolution, Case report, *C. davisae* implant-associated bone infection

## Abstract

**Background:**

Data on antimicrobial resistance mechanisms are scanty for *Cedecea spp.*, with very variable antibiotic resistance patterns documented. Here we report the first in vivo resistance evolution of a *C. davisae* clinical isolate in a patient with a complex hand trauma and provide insight in the resistance mechanism, leading to therapeutic implications for this pathogen.

**Case presentation:**

*Cedecea davisae* was isolated from a patient with hand trauma during a first surgical debridement. Six days after primary surgical treatment and under antimicrobial treatment with amoxicillin-clavulanic acid and later cefepime, follow up cultures yielded *C. davisae* which demonstrated a resistance development. The susceptible parental isolate and its resistant derivative were characterized by whole genome sequencing, *ampC, ompC and ompF* by RT- PCR. The resistant derivative demonstrated an A224G SNP in *ampD*, the transcriptional regulator of *ampC*, leading to a His75Arg change in the corresponding AmpD protein. AmpC transcription of the resistant derivative was 362-times higher than the susceptible isolate. Transcription levels of *ompF* and *ompC* were 8.5-fold and 1.3-fold lower, respectively, in the resistant derivative. Downregulation of OmpF putatively resulted from a mutation in the presumed promoter region upstream of the dusB-Fis operon, a proposed regulator for *ompF*.

**Conclusions:**

This case demonstrates the in vivo resistance development of *C. davisae* within 7 days similar to that of the members of the *Enterobacter cloacae* complex. Our findings add valuable information for future therapeutic management of these opportunistic pathogens as they warrant the same empirical treatment as AmpC producers.

## Background

*Cedecea* spp. are Gram-negative bacilli belonging to the *Enterobacterales* [1]. They can act as opportunistic pathogens, principally in immunocompromised hosts, with *C. davisae, C. lapagei* and *C. neteri* all documented as having clinical significance [2]. Although infections are infrequent and sporadic, reports are increasing [2]. Recent papers indicate 13 case reports of *C. davisae* infections to date, starting from 1977. Infection sites include blood, sputum, gall bladder, skin wounds and abscesses [2, 3]. More than three quarters of the patients were ≥ 50 years of age, and most were severely immunocompromised, with multiple comorbid diseases [2]. Very variable antibiograms have been documented for the genus: resistances to amoxicillin, amoxicillin-clavulanate and cephalosporins are frequent, though not universal [2]. Data on resistance mechanisms are scarce. Acquired New Delhi metallo-β-lactamase-1 (NDM-1) has been detected in *C. lapagei* and *C. davisae* [2, 3]*.* Perhaps of greater general significance, a novel AmpC β-lactamase was characterized from a *C. davisae* clinical isolate in 2014 [3]; this resembled the chromosomal AmpC β-lactamases of *Enterobacter* spp. and was non-transferable.

Here we report in vivo evolution of β-lactam resistance in a *C. davisae* implant-associated bone infection, characterized by whole genome sequencing. Expression of *ampC, ompC and ompF* was assayed by Reverse Transcriptase PCR (RT-PCR). Our findings add valuable information for future therapeutic management of these opportunistic pathogens.

## Case presentation

**Fig. 1:**
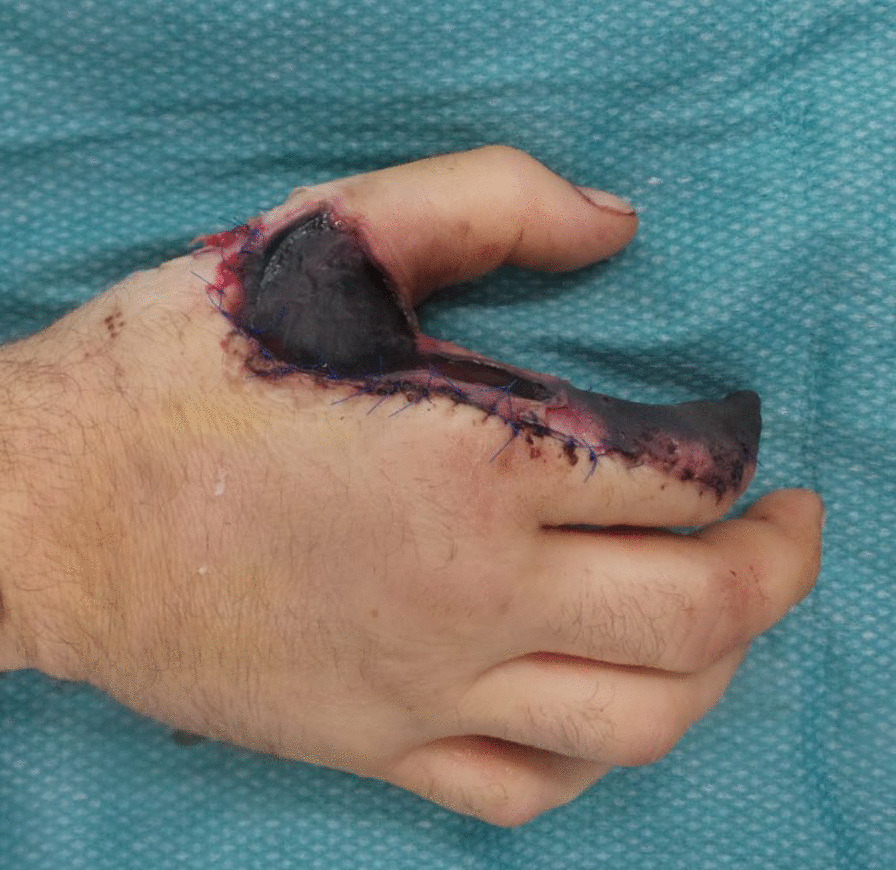
Clinical presentation at the emergency room at our hospital

A 33-year-old man with a history of curatively-treated seminoma presented to our emergency room with skin and soft tissue necrosis on his right hand, along with increasing pain, 1 day after being discharged from an external hospital (Fig. 1). Two weeks previously he had suffered a complex right-hand trauma while cleaning an industrial flour mixer. The external hospital had immediately performed an initial surgery, involving osteosynthesis and tendon repair. Due to a type III open fracture he had received an empirical treatment with amoxicillin-clavulanic acid (6 g/day i.v.) until discharge.

**Fig. 2:**
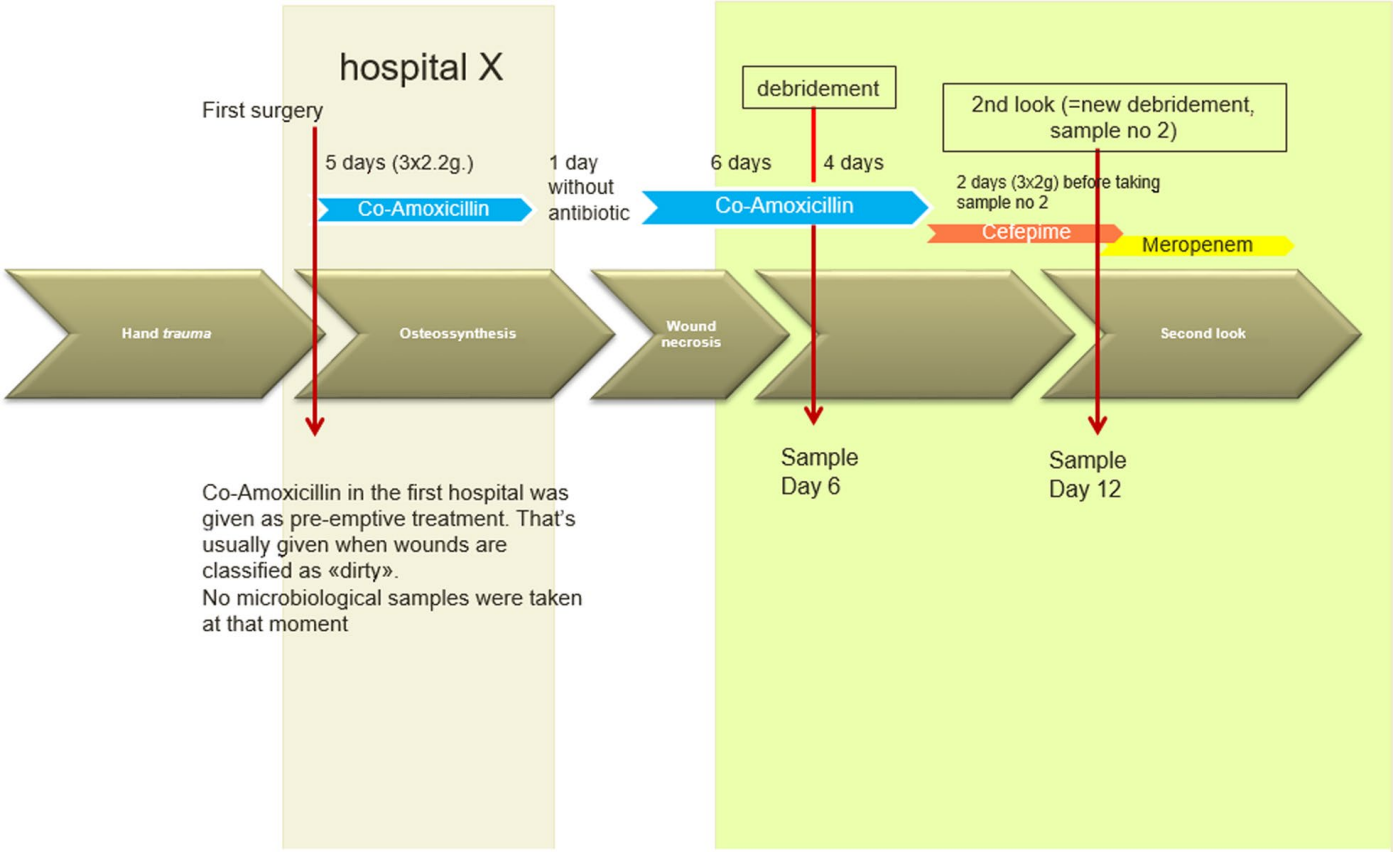
Isolation of C. davisae during Course of antibiotic and surgical treatment

Following presentation at our hospital amoxicillin-clavulanic acid (6 g/day i.v.) was restarted; it was assumed that the tissue necrosis was caused by poor blood circulation (Fig. 1). Since the patient’s symptoms did not improve, debridement, necrosectomy and transmetacarpal amputation of the index finger and partial removal of osteosynthesis material were performed 6 days after presentation (Day 6, Fig. 2). Amoxicillin-clavulanic acid was continued for 4 days post-surgery, until samples, taken on the day of surgery, revealed the growth of *C. davisae* resistant to this agent (Table 1). Anaerobic cultures were also performed and yielded no growth. Antimicrobial treatment was then switched to cefepime (6 g/d i.v.), based on a concern that *C. davisae* might have a potential to overexpress an AmpC enzyme. Two days after switch to cefepime, a new “second-look” debridement surgery was performed (Day 12, Fig. 2). Cultures at this time again yielded *C. davisae* but with additional resistance to ceftriaxone, ceftazidime, piperacillin-tazobactam and a raised ‘on the breakpoint’ MIC for ertapenem (0.5 mg/L, Table 1). While precise MIC data for cefepime were pending, antibiotic therapy was switched to meropenem (3 g/day i.v.) and a reconstruction using a radial forearm flap was undertaken to close the defect and cover the exposed bone and remaining ostheosynthesis material. Subsequent testing showed that the cefepime MIC for the strain had also increased, though only from 0.047 to 1 mg/L.

**Table 1:** Phenotypic susceptibility patterns of the two *C. davisae* isolates

Antibiotic	Parental isolate (Day 6:18.11.20)	Resistant isolate (Day 12: 24.11.20)
Amoxicillin-clavulanic acid	32 (R)	> 64 (R)
Piperacillin-tazobactam	≤ 6 (S)	32 (R)
Cefotaxime	≤ 0.5 (S)	> 16 (R)
Ceftazidime	≤ 0.5 (S)	> 16 (R)
Cefepime	< 1; 0.047^a^ (S)	< 1; 1 ^a^ (S)
Aztreonam	≤ 1(S)	> 8 (R)
Imipenem	≤ 0.5 (S)	≤ 0.5 (S)
Meropenem	≤ 0.5 (S)	≤ 0.5 (S)
Ertapenem	≤ 0.19 (S)	0.5 (S)
Ciprofloxacin	≤ 0.19 (S)	≤ 0.19 (S)
Co-Trimoxazole	≤ 0.25 (S)	≤ 0.25 (S)
Gentamicin	≤ 0.5 (S)	≤ 0.5 (S)
Tobramycin	≤ 0.5 (S)	≤ 0.5 (S)

S, susceptible, R, resistant according to EUCAST guidelines (version 10)

^a^By Etest

The patient thereafter showed a satisfactory course and was 2 weeks later released into outpatient care with oral trimethoprim-sulfamethoxazole (3 g/d) for further 6 months. There were no signs of a recurrent infection 4 weeks after stopping the antibiotic therapy. The plan is to remove the remaining osteosynthesis material in a further surgery and to treat the underlying osteomyelitis with ciprofloxacin (Fig. 2).

### Microbiological testing

All samples from the patient were processed in November 2020 according to the accredited routine procedures of the Centre for Laboratory Medicine in St. Gallen, Switzerland. Identification was with MALDI-ToF mass spectrometry (Bruker Daltonics, Bremen, Germany) using the BDAL 9.0 database; routine susceptibility testing was performed with the NMIC-417 panel on the BD Phoenix™ M50 (Becton Dickinson, Franklin Lakes, NJ, USA). Further broth microdilution testing using Sensititre GNX2F plates (Trek Diagnostic Systems, UK) with Mueller–Hinton broth (BBL, Becton Dickinson) was performed at the Medical Research Centre. In the case of cefepime, precise ‘on-scale’ MICs were determined by Etest (bioMérieux, Marcy l’Etoile, France). Antimicrobial susceptibility data were interpreted according to EUCAST guidelines (version 10.0, 2020 [4]).

### Whole genome sequencing and mutation analysis

The Day 6 isolate and its resistant Day 12 counterpart were characterized by whole genome sequencing (WGS). DNA extraction was performed using the QIAsymphony DSP DNA Mini Kit (QIAGEN GmbH, Hilden, Germany); sequencing with an Illumina MiSeq instrument and the Nextera XT library preparation kit (Illumina Inc., USA); all were used according to the manufacturers’ procedures. Assembly was performed using the Ridom Seqsphere + Software with standard settings (Ridom: Munster, Germany). Both genomes had over 40× coverage (NCBI accession numbers: SAMN18652104 and SAMN18652105). Annotation was performed using the Prokka software (version 1.14.6) [5]; For SNP detection, the susceptible parental isolate was used as a reference, and calling was conducted using Snippy (version 4.6.0) [6].

### Evaluation of transcription levels

**Fig. 3:**
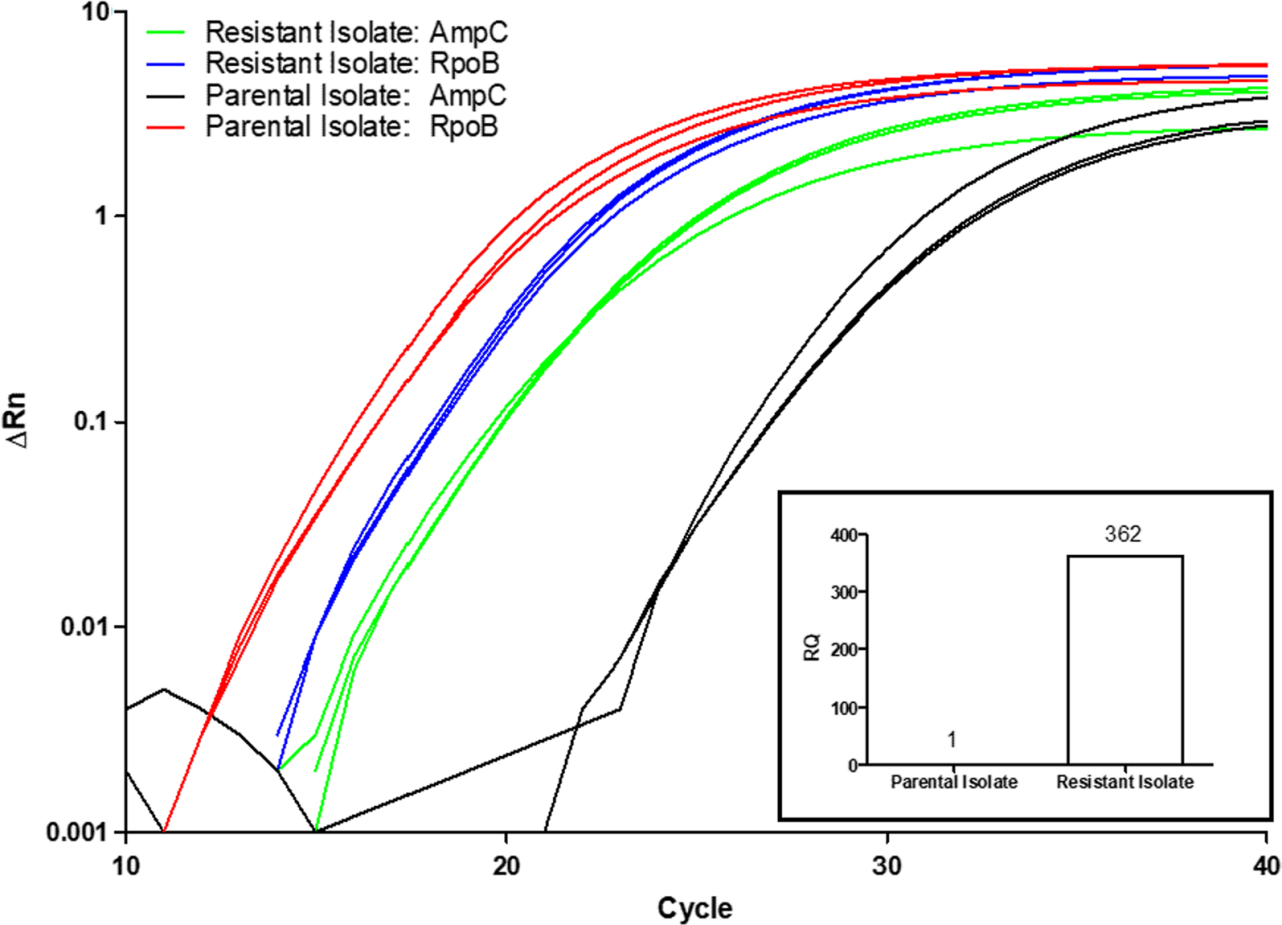
bla_AmpC_ RT-PCR Amplification plot with rpoB as reference. Fold change of 362 calculated with 2^−ΔΔCT^ Method

Reverse transcriptase (RT)-PCR was used to measure mRNA levels for *bla*_*AmpC*_, *ompF* and *ompC* (Fig. 3), using the primers listed in Table 2*.* Mid-logarithmic phase cultures (0.5 ml) of the Day 6 and 12 *C. davisae* isolates were treated with the RNAprotect reagent (Qiagen). RNA was then extracted with an RNeasy Mini Kit (Qiagen) and the eluate treated with DNase I (Qiagen), used according to the manufacturer’s instruction. RT-PCR was subsequently performed using the Power SYBR®Green RNA-to-CT 1-Step Kit (Thermo Fisher Scientific, Vilnius, Lithuania) and a QuantStudio 5 Real-Time PCR System (Applied Biosystems by Thermo Fisher Scientific) at an annealing temperature of 60 °C. Transcript measurements were carried out in triplicate and measurements were repeated twice. Quantification of relative target gene expression was by the 2^−ΔΔCT^ method, using *rpoB* as a reference, as described previously [7]. The original Day 6 *C. davisae* isolate was used as the calibrator (Table 2).

**Table 2:** Primers used for RT-PCR expression analysis

Name	Sequence	Reference
RT Ceda RpoB_F2	5' TGA CAA GCT CGA CAA ACT GC 3'	This study
RT Ceda RpoB_R2	5' CGC CCT GAG TGA TTT TAC GG 3'	This study
RT Ceda AmpC_F1	5' AGT GCT GGA ACC ATT GAA GC 3'	This study
RT Ceda AmpC_R1	5' TTC GAT GCT GGA CTT AAC GC 3'	This study
RT Ceda OmpC_F2	5' TGT TAC CTG CGG CAT CAT TG 3'	This study
RT Ceda OmpC_R2	5 'GCT ATG AGT CCC AGG GCT TT 3'	This study
RT Ceda OmpF_F2	5' CCG TAC CAA TGC CCA ACA AA 3'	This study
RT Ceda OmpF_R2	5' AGT GCT GCC AGG TAG ATG TT 3 '	This study

### Microbiological results

Susceptibility data for the Day 6 and Day 12 isolates are summarized in Table 1. Both isolates were resistant to amoxicillin/clavulanate and both susceptible to cefepime, imipenem, meropenem and various non-β-lactams. They differed in that the Day 6 isolate was susceptible to ceftriaxone, ceftazidime and piperacillin/tazobactam whereas the Day 12 isolate was resistant to these agents and had reduced susceptibility to ertapenem. The cefepime MIC for the Day 12 isolate, by Etest, was 21-fold higher than for the Day 6 isolate (1 mg/L vs. 0.047 mg/L, Table 1) but remained in EUCAST’s susceptible range [4]. Except for SNPs, detailed below, the two isolates were identical by WGS, confirming that they represented the same strain.

The Day 12 derivative had an A224G SNP in *ampD*, the transcriptional regulator of *ampC*, leading to a His75Arg change in the corresponding AmpD protein. Correlating with this, AmpC transcription in the resistant derivative was 362-times higher than the Day 6 isolate (Fig. 3). There were no mutations within *ompF* and *ompC*; however, transcription levels of these outer membrane proteins were 8.5-fold and 1.3-fold lower, respectively, in the resistant derivative.

**Fig. 4:**
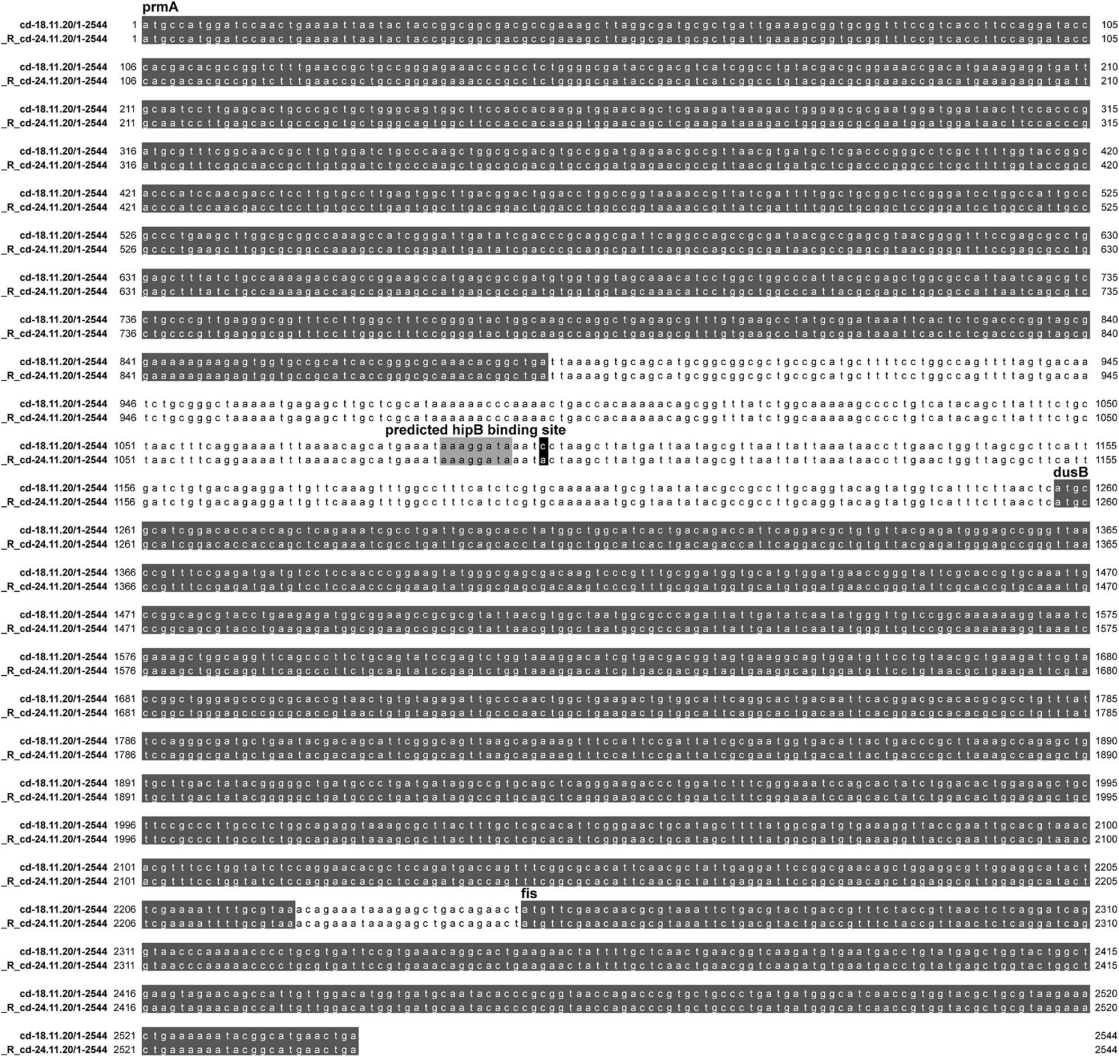
C→A SNP detected in the promoter rich intergenic region upstream of dusB gene. Sequence alignment of the region between prmA and fis genes was performed with MAFFT [13] and visualized and annotated in Jalview v2.11.1.4 [14]

Six further SNPs distinguished the parent and the resistant organisms, potentially explaining these latter differences. Three of these SNPs were in intergenic regions (Table 3) and one (C→A) was 162 nucleotides upstream of *dusB*, which belongs to the *dusB-fis* operon, where Fis is a transcriptional regulator reported to affect expression of *ompF* [8]. Notably, this SNP was located in a potentially promoter-rich intergenic region, four nucleotides downstream of a predicted helix-turn-helix transcription factor *hipB* binding site, as found using the Softberry [9] (Fig. 4).

**Table 3:** SNPs between the parent (Day 6) and the resistant (Day 12) isolates

Contig	Position	Day 6	Day 12	Effect	Gene	Product
5	184001	A	G			
11	91073	T	C	Missense_variant A224G p.His75Arg	*ampD*	1;6-anhydro-N-acetylmuramyl-l-alanine amidase AmpD
13	36350	A	G	Missense_variant T614C p.Val205Ala	*yicL*	Putative inner membrane transporter YicL
35	57393	A	G	Missense_variant T1645 > C p.Cys549Arg	*hemR*	Hemin receptor
41	87395	CCCC	TCCA	Missense_variant 602_605delCCCCinsTCCA p.ThrPro201IleHis	*mdoC*	Glucan biosynthesis protein C
41	576365	G	A			
54	159944	C	A			Intergenic region upstream of *dusB* gene

p. corresponding amino acid change

NCBI Accession Numbers: parent isolate (day 6): SAMN18652104; resistant isolate (day 12): SAMN18652105

## Discussion and conclusion

This case demonstrates that resistance to β-lactams can develop in *C. davisae* via mutation of *ampD*, leading to hyperproduction of the AmpC β-lactamase, as also frequently occurs e.g. in members of the *Enterobacter cloacae* complex [10]. Although AmpC inducibility was not investigated, an *ampR* homologue was found by sequencing upstream the *ampC*, and an *ampR-ampC* operon, predicting inducibility and the increased risk of selecting hyperproducers [11], has been described previously in the related species, *C. neteri* [12]. We suggest that the additional rise in ertapenem MIC seen here reflected downregulation of OmpF, putatively as a result of mutation in the presumed promoter region upstream of the dusB-Fis operon, a proposed regulator for OmpF.

Resistance to β-lactams, including carbapenems, has been associated previously with a combination of AmpC activity and loss of both porins OmpC and OmpF in *C. davisae* [3] but the in-vivo evolution of resistance associated with these mechanisms has not been recorded in the literature. It is perhaps surprising that this evolution occurred with sequential use of amoxicillin-clavulanate acid, which lacked activity against even the initial isolate, and cefepime, which retained activity even against the second isolate, albeit with a raised MIC. Our findings should inform future therapeutic management of infections due to these uncommon opportunistic pathogens, underscoring that they warrant the same caution as other species where AmpC derepression is a hazard.

## Data Availability

Whole genome sequences of the isolates are available on NCBI Accession Numbers: SAMN18652104 and SAMN18652105.

## Abbreviations

MALDI-ToF: Matrix-assisted laser desorption/ionization time-of-flight-mass spectrometry
MIC: Minimal inhibitory concentration
NDM-1: New Delhi metallo-β-lactamase-1
RT-PCR: Reverse Transcriptase PCR
SNP: Single nucleotide polymorphism
WGS: Whole genome sequencing
